# Cerebrovascular complications in patients with community-acquired bacterial meningitis: occurrence and associated factors in the COMBAT multicenter prospective cohort

**DOI:** 10.1186/s12879-023-08320-x

**Published:** 2023-06-05

**Authors:** Amine Benadji, Thomas Debroucker, Guillaume Martin-Blondel, Laurent Argaud, Virginie Vitrat, Charlotte Biron, Michel Wolff, Bruno Hoen, Xavier Duval, Sarah Tubiana

**Affiliations:** 1 Inserm Clinical Investigation Center 1425, Bichat Hospital, APHP, 46, rue Henri Huchard, Paris, 75018 France; 2 Department of Neurology, Pierre-Delafontaine Hospital, Saint-Denis, France; 3 Department of Infectious Diseases, Toulouse Institute for Infectious and Inflammatory Diseases, University Hospital of Toulouse, INSERM UMR1291 - CNRS UMR5051 - Université Toulouse III, Toulouse, France; 4 Medical Intensive Care Unit, Hospices Civils de Lyon, Edouard Heriot Hospital, Lyon, France; 5 Lyon University, INSERM UMR1060 (CarMeN), Lyon, France; 6 Department of infectious diseases, Annecy Genevois Hospital, Annecy, France; 7 Center for the Prevention of Infectious and Transmitted Diseases of the UHC of Nantes, Nantes, France; 8 Department of Infectious Diseases, INSERM, University Hospital Center of Nantes, Nantes, CIC 1413 France; 9 Neuro-surgical Intensive Care Unit, Saint-Anne Hospital, Paris, France; 10 Department of infectious diseases, University Hospital of Nancy, Vandoeuvre-lès-Nancy, France; 11 Université Paris Cité, INSERM, Infection, Antimicrobials, Modelling, Evolution (IAME), Paris, France

**Keywords:** Community-acquired meningitis, Cerebrovascular complications, Dexamethasone

## Abstract

**Background:**

Community-acquired bacterial meningitis is a rare but severe central nervous system infection that may be associated with cerebrovascular complications (CVC). Our objective is to assess the prevalence of CVC in patients with community-acquired bacterial meningitis and to determine the first-48 h factors associated with CVC.

**Methods:**

We analyzed data from the prospective multicenter cohort study (COMBAT) including, between February 2013 and July 2015, adults with community-acquired bacterial meningitis. CVC were defined by the presence of clinical or radiological signs (on cerebral CT or MRI) of focal clinical symptom. Factors associated with CVC were identified by multivariate logistic regression.

**Results:**

CVC occurred in 128 (25.3%) of the 506 patients in the COMBAT cohort (78 (29.4%) of the 265 pneumococcal meningitis, 17 (15.3%) of the 111 meningococcal meningitis, and 29 (24.8%) of the 117 meningitis caused by other bacteria). The proportion of patients receiving adjunctive dexamethasone was not statistically different between patients with and without CVC (p = 0.84). In the multivariate analysis, advanced age (OR = 1.01 [1.00-1.03], p = 0.03), altered mental status at admission (OR = 2.23 [1.21–4.10], p = 0.01) and seizure during the first 48 h from admission (OR = 1.90 [1.01–3.52], p = 0.04) were independently associated with CVC.

**Conclusions:**

CVC were frequent during community-acquired bacterial meningitis and associated with advanced age, altered mental status and seizures occurring within 48 h from admission but not with adjunctive corticosteroids.

**Supplementary Information:**

The online version contains supplementary material available at 10.1186/s12879-023-08320-x.

## Introduction

Community-acquired bacterial meningitis was a rare central nervous system infection with an annual incidence around 2/100 000 inhabitants, affecting all age groups and responsible for high morbidity and mortality [1–3]. Pneumococcal and meningococcal bacteria are the microorganisms responsible for 85% of meningitis in adults in industrialized countries [2–5]. The benefit of early adjunctive dexamethasone differs in the literature according to age, microorganisms, and to the level of medical care of the country. Randomized controlled trials and meta-analyses have shown that dexamethasone therapy given during the 4 first day of antibiotic reduces neurological sequelae and death in adults with bacterial meningitis caused by *S. pneumonia* [6–8]. Cohort studies have shown improved outcomes of bacterial meningitis after implementation of adjunctive dexamethasone therapy in several countries [3, 5, 9]. No beneficial effects of adjunctive corticosteroids have been identified in studies done in low-income countries [8].

Cerebrovascular complications (CVC) are common in patients with meningitis with reported rates ranging from 10 to 29% and their occurrence influences patients morbidity and mortality [10–12]. The predominant pathophysiological mechanism of CVC is localized cerebral vasculitis [10, 11], which leads to the activation of coagulation and the inhibition of fibrinolysis, causing thrombosis, infarction and / or hemorrhage [12–15]. Rarer reported mechanisms are vasospasms, or disseminated cerebral intravascular coagulation; septic emboli in patients having both meningitis and endocarditis have also been reported [16–18]. The role of corticosteroids in favoring the occurrence of CVC has been discussed in one study, which reported the occurrence of delayed cerebral thrombosis in patients treated by early adjunctive dexamethasone [19].

The aim of this study was to assess in the prospective multicenter French COMBAT cohort, the prevalence of CVC in patients with community-acquired bacterial meningitis and to determine the first-48 h factors associated with CVC including the use of corticosteroids.

## Methods

### Population and study design

Our study is based on data collected from the prospective French multicenter cohort (COMBAT) conducted in 69 hospitals in metropolitan France and overseas territories between February 2013 and July 2016 which included between February 2013 and July 2015 consecutive adult patients with community-acquired acute bacterial meningitis [20]. In the present ancillary analysis, we investigated cerebrovascular complications. We excluded from this analysis patients with both infective endocarditis and meningitis since pathophysiological mechanism of CVC is different, resulting from the migration of an infectious embolus from the cardiac valve lesion [21].

### Patient and microbiological characteristics

Demographic data, predisposing conditions, concomitant infections, initial symptoms, intra-hospital complications, use for corticosteroid and cerebral imaging were prospectively collected in an electronic CRF. Immunosuppressed patients with congenital or acquired immunodeficiency, asplenic patients, patients under immunosuppressive drugs and patients with HIV infection were considered immunocompromised. Data including CSF culture, blood culture, and pathogen identification were collected. Microbiological analyzes were carried out locally in hospital microbiology laboratories and bacterial strains were also sent to the national reference center for each corresponding microorganism.

### Cerebrovascular complications (CVC) definition

CVC were defined by the presence of clinical or radiological signs (on cerebral CT or MRI) of focal clinical symptom. The focal clinical symptoms considered were the following: motor impairment, cerebellar syndrome, visual impairment (occulomotor paralysis or visual field amputation), sensory impairment (deep pain sensibility, tactile and protopathic sensibilities), aphasia, and pyramidal syndrome. A pyramidal syndrome was defined as an unilateral weakness (hemiparesia or hemiplegia) including or not lower facial palsy, with or without brisk reflexes, hypotonia or spasticity, with or without an ipsilateral Babinski’s sign.

Patients were considered as having radiological signs of CVC when cerebral imaging (cerebral CT or MRI) disclosed ischemic and/or hemorrhagic lesions associated or not with vasculitis and/ or thrombophlebitis. CVC were hereafter referred to as 1/ “Definite symptomatic CVC”, 2/ “Definite asymptomatic CVC” and 3/ “Probable CVC”. Definite symptomatic CVC corresponds to situations where patients had both focal clinical symptom and radiological signs of CVC, definite asymptomatic CVC when the patients had no focal clinical symptom but radiological signs of CVC and probable CVC when the patients only showed focal clinical symptom, without confirmation by radiological signs. In these latter patients, CT scanning was either not performed, or was performed but did not show any radiological signs supporting the diagnosis of CVC, as defined above [12]. CVC were divided into those occurring within 48 h from admission and those occurring after.

### Statistical analysis

The explanatory variables were chosen from those reported in the literature and from the supposed pathophysiology of CVC. We considered only the variables present at baseline and during the first 48 h after lumbar puncture. A descriptive analysis of the clinical, microbiological and therapeutic characteristics of the patients was performed. Quantitative variables were expressed as medians and interquartile range and the categorical variables were summarized as counts (percentage). A bivariate analysis was performed to investigate the factors associated with CVC. Discrete and categorical variables were compared using chi square test or the Fisher exact test as appropriate. Continuous variables were expressed as median (IQR) and differences were tested with the independent t-test for normally distributed variables or the Mann-Whitney U test otherwise.

Multivariate analysis was performed by a logistic regression model. Variables which degree of bivariate significance was below 0.20 were initially introduced into the model. The final model retained only variables which significance level was less than or equal to 0.05. We assessed the linearity of the association between continuous variables and outcome with the Lemeshow goodness of fit and by visual inspection. In order to better analyze the role of corticosteroid initiation, a sensitivity analysis was performed excluding patients with symptomatic CVC already present at hospital admission or occurring during the 48 first hours. Statistical analyzes were performed using the STATA Version 13 software and variables with a p statistic less than 0.05 were considered statistically significant.

## Results

### Patient characteristics

**Fig. 1:**
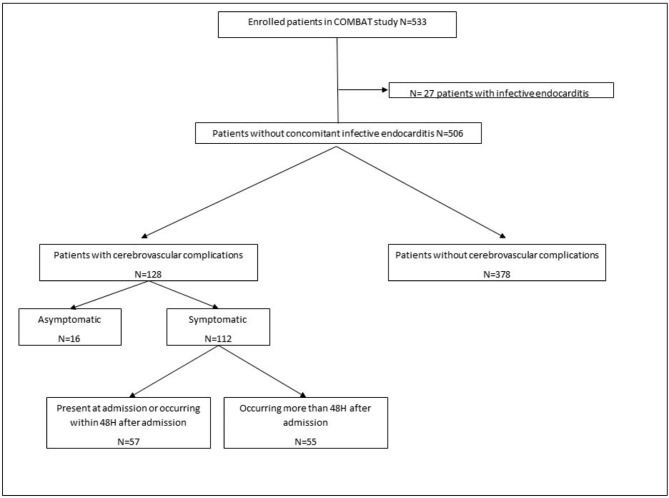
Study flow chart

Among the 533 patients included in the COMBAT study between February 2013 and July 2015, 27 patients were excluded because they had concomitant endocarditis; the remaining 506 patients were analyzed for the present ancillary study (Fig. 1).

Patient characteristics are presented in Table 1. Median age was 58.4 (42.0-68.5) years and 54.3% were men (275/506). Concomitant infection such as otitis and/or sinusitis were present in 147 (29.8%) patients; an immunocompromised state was present in 129 (25.8%) patients. The most frequent clinical presentations of meningitis were headaches (71.5%), altered mental status (71.4%), fever (67.6%) and neck stiffness (63.2%). After hospital admission, the main complications during the first 48 h were severe sepsis in 175 (35.5%) patients, seizures in 51 (10.3%) patients. Brain imaging (CT scan or MRI) was performed in 433 (87.8%) patients. Adjunctive dexamethasone was administered in 358 (72.4%) patients. Identification of microorganism based on blood or CSF culture results yielded *S. pneumoniae* in 265 (53.8%) patients, *N. meningitidis* in 111 (22.5%) patients, and other microorganisms in 117 (23.7%) patients.

**Table 1:** Socio-demographic, clinico-biological and radiological characteristics of the 506 patients of COMBAT study

Characteristics	n (%) or Median (Q1-Q3)
**Demographic data**	
Age, years	58.4 (42-68.5)
Male sex	275/506 (54.3)
**Predisposing conditions**,	
Immunocompromised state*	129/500 (25.8)
Diabetes mellitus	71/500 (14.2)
Chronic alcoholism	74/500 (14.8)
Active smoking	107/500 (21.4)
**Symptoms at presentation**	
Headache	347/485 (71.5)
Nausea and/or vomiting	254/506 (50.2)
Fever (≥ 38 °C)	342/506 (67.6)
Neck stiffness	308/487 (63.2)
Altered mental status (Glasgow score < 14)	354/496 (71.4)
**Concomitant infections**	
Otitis/sinusitis	147/494 (29.8)
Pneumonia	52/494 (10.6)
**Blood chemistry tests**	
Leukocyte count (103 cells/mm^3^) (N = 484)	15.2 (9.0–21.2)
**Indexes of inflammation in the CSF**	
Leukocyte count (cells/mm^3^) (N = 497)	1530 (332–5000)
CSF Protein (g/l) (N = 484)	4.1 (2.0–6.6)
CSF/ blood glucose ratio (mmol/l) (N = 484)	0.6 (0.1–2.5)
**Intra-hospital complications in the first 48 h**	
Seizure	51/494 (10.3)
Severe Sepsis**	175/493 (35.5)
Extensive necrotic purpura	48/497 (9.7)
Disseminated intravascular coagulation	33/492 (6.7)
Brain abscess	32/487 (6.6)
**Brain imaging performed during hospitalization**	
CT scan	414/492 (84.2)
MRI	184/490 (37.5)
CT scan or MRI	433/493 (87.8)
**Adjunctive dexamethasone**	
Administered before antibiotic initiation	53/494 (10.7)
Administered at the same time as antibiotics	180/494 (36.4)
Administered after antibiotic initiation	125/494 (25.3)
No dexamethasone administered during hospitalization	136/494 (27.6)
**Microorganisms**	
*Streptococcus pneumoniae*	265/493 (53.8)
*Neisseria meningitidis*	111/493 (22.5)
Other microorganisms ***	117/493 (23.7)

CSF: Cerebrospinal fluid;

* An immunosuppressive state was defined by the use of immunosuppressive drugs, the presence of asplenia, congenital immunodepression, acquired hypogammaglobulinemia, hepatic cirrhosis, metastatic cancer, chronic hematological malignancy, or HIV infection

**according to the 2001 International Sepsis Conference definition for severe sepsis (i.e., sepsis and lactates > 4 mmol/L or hypotension before fluid resuscitation or ≥ 1 organ dysfunction [respiratory: PaO2/FiO2 < 300; or renal: serum creatinine > 176 µmol/L; or coagulation: INR > 1.5; or liver: INR > 4, bilirubin > 78 µmol/L; or thrombocytopenia: <105/mm3; or cognitive functions: <13 on Glasgow Coma Scale])

*** *Listeria monocytogenes* in 32/493 (6.5%), *Haemophilus influenzae* or related in 25/493 (5.1%), Non pneumococcal streptococcus in 32/493 (6.5%), Escherichia coli in 7/493 (1.4%), *Staphylococcus aureus* in 4/493 (0.8%), *Mycobacterium tuberculosis* complex in 2/493 (0.4%), other microorganisms in 15/493 (3.0%)

### Cerebrovascular complications

Out of the 506 patients, 128 (25.3%) were classified as having cerebrovascular complications: “Definite symptomatic CVC” in 16 (3.2%) patients, “Definite asymptomatic CVC” in 16 (3.2%) patients and “Probable CVC” in 96 (19.0%) patients (Table 2). Among the 433 patients with brain imaging, 32 (7.4%) had abnormal findings: intracerebral hemorrhage in 6 (1.4%) patients, ischemic stroke in 27 (6.2%) patients and cerebral thrombosis in 2 (0.5%) patients.

**Table 2:** Characteristics of the 128 patients with cerebrovascular complications in the COMBAT study

	n (%)
**Cerebrovascular complications**	128/506 (25.3%)
Definite symptomatic	16/506 (3.2)
Definite asymptomatic	16/506 (3.2)
Probable	96/506 (19.0)
**Focal clinical symptom**	112/128 (87.5)
Motor impairment and / or cerebellar syndrome	74/128 (57.8)
Visual impairment	10/128 (7.8)
Sensory impairment	12/128 (9.4)
Aphasic disorder	44/128 (34.4)
Pyramidal syndrome	5/128 (3.9)
**Radiological signs of CVC**	
Abnormal imaging on CT scan or MRI	32/433 (7.4)
Intracerebral hemorrhage	6/433 (1.4)
Ischemic stroke	27/433 (6.2)
Cerebral thrombosis	2/433 (0.5)

CVC: Cerebrovascular complications

Ninety-six (75%) patients had “probable CVC “. Among the 128 patients with CVC, 112 (87.5%) patients presented focal clinical symptom (motor impairment and / or cerebellar syndrome in 74 (57.8%) patients, visual impairment in 10 (7.8%) patients, sensory impairment in 12 (9.4%) patients, aphasia in 44 (34.4%) patients, and pyramidal syndrome in 5 (3.9%) patients. Cranial imaging was performed in 125 out of 128 (97.6%) patients with CVC whereas it was performed in 308 (84.4%) patients without CVC (p < 0.001). Overall, 55 out of the 128 CVC occurred more than 48 hours after admission (Fig. 1) with no significant difference in the timing between microorganisms (pneumococcal meningitis in 35/57 (61.4%) patients with CVC occurring within 48 hours after admission versus 35/55 (63.6%) in patients with CVC occurred more than 48 hours after admission).

According to the microorganism, 78 (29.4%) of the 265 patients with pneumococcal meningitis developed CVC, 17 (15.3%) of the 111 meningococcal meningitis, and 29 (24.8%) of the 117 meningitis caused by other bacteria.

### Factors associated with CVC

In the bivariate analysis, patients with CVC were older than those without CVC (median age, 62 years [IQR 51–72] vs. 56 [IQR 38–67], P < 0.001). Concomitant infections such as otitis or sinusitis (32% vs. 28.9%; p = 0.57), pneumonia (13.2% vs. 9.6%; p = 0.24) and immunocompromised status (30.5% vs. 24.2%; p = 0.15) were not significantly different between the two groups. On the contrary, patients with CVC had more frequently altered mental status (85.8% vs. 66.4%; p < 0.001), and seizures during the first 48 h of hospitalization (17.5% vs. 7.9%; p = 0.004) (Table 3).

**Table 3:** Factors associated with cerebrovascular complications in the COMBAT study; N = 506

	Patients with CVC (N = 128)	Patients without CVC (N = 378)	P-value of bivariate analysis	Multivariable odds ratio [95% CI]	P-value of multivariable analysis
**Demographic data**					
Age, years (Median, IQR)	62 (51–72)	56 (38–67)	< 0.001	1.01 [1.00–1.03]	0.03
Male	70/128 (55.0%)	205/378 (54.0%)	1.00		
**Predisposing conditions, concomitant infections**					
Otitis/sinusitis	41/128 (32.0%)	106/366 (28.9%)	0.57		
Pneumoniae	17/128 (13.2%)	35/366 (9.6%)	0.24		
Immunocompromised state*	39/128 (30.5%)	90/372 (24.2%)	0.15		
**Symptoms on presentation**					
Altered mental status	109/127 (85.8%)	245/369 (66.4%)	< 0.001	2.23 [1.21–4.10]	0.01
**Blood chemistry tests**					
Leukocyte count (10^3^ cells/mm^3^)	14.9 (8.6–20)	15.3 (9.1–21.5)	0.41		
**Indexes of inflammation in the CSF**					
Leukocyte count (cells/mm^3^)	1105 (250–4050)	1760 (367–5700)	0.14		
Protein (g/l)	5.7 (3.0–8.4)	3.9 (2.0–6.3)	< 0.001		
CSF: blood glucose ratio (mmol/l)	0.2 (0.05–1.6)	0.8 (0.1–2.55)	0.03		
**Intra-hospital complication in the first 48 h**					
Seizure	22/126 (17.5%)	29/368 (7.9%)	0.004	1.90 [1.01–3.52]	0.04
Extensive necrotic purpura	8/128 (6.2%)	40/369 (10.8%)	0.16		
Disseminated intravascular coagulation	8/127 (6.3%)	25/365 (6.8%)	1.00		
**Adjunctive dexamethasone**					
Administered before antibiotic initiation	13/127 (10.2%)	40/367 (10.9%)	0.84		
Administered at the same time as antibiotics	50/127 (39.4%)	130/367 (35.4%)			
Administered after antibiotic initiation	29/127 (22.8%)	96/367 (26.2%)			
No dexamethasone administered during hospitalization	35/127 (27.6%)	101/367 (27.5%)			
**Brain imaging performed**	125/128 (97.6%)	308/365 (84.4)	< 0.001		
**Microorganisms**					
*Streptococcus pneumoniae*	78/124 (62.9%)	187/369 (50.7%)	< 0.001		
*Neisseria meningitidis*	17/124 (13.7%)	94/369 (25.5%)			
Other microorganisms*	29/124 (23.4%)	88/369 (23.8%)			

* An immunosuppressive state is defined by the use of immunosuppressive drugs, the presence of asplenia, congenital immunodepression, acquired hypogammaglobulinemia, hepatic cirrhosis, metastatic cancer, malignant hemopathy, chronic or HIV infection

** other microorganisms represented by *Listeria monocytogenes* in 7/124 (5.5%) vs. 25/369 (6.6%), *Haemophilus influenzae* or related in 2/124 (1.6%) vs. 23/369 (6.2%), non-pneumococcal streptococcus in 11/124 (8.9%) vs. 21/369 (5.7%), *Escherichia coli* in 3/124 (2.4%) vs. 4/369 (1, 0%), *Staphylococcus aureus* in 1/124 (0.8%) vs. 3/369 (0.8%), *Mycobacterium tuberculosis* complex in 1/124 (0.8%) vs. 1/369 (0.3%), other microorganisms at 4/124 (3.2%) vs. 11/369 (3%)

There was no difference in median CSF leukocyte count between both groups (1105 per mm^3^ [IQR 250–4050] vs. 1760 [IQR 367–5700]; p = 0.14) but CSF protein was higher in patients with CVC (5.7 g/l [IQR 3.0-8.4] vs.3.9 g/l [IQR 2.0–6.3]; p < 0.001). The distribution of microorganisms was different in both groups (p < 0.001) (Table 3): *S. pneumoniae* was more frequently the causing microorganism in patients with CVC (62.9% vs. 50.7%). Early Adjunctive treatment with dexamethasone was widely used (72.4%), with no significant difference between the two groups (p = 0.84), regardless of whether dexamethasone was administered before, at the same time, or after initiation of antibiotics (Table 3).

In the multivariate analysis, advanced age (OR = 1.01; 95% CI [1.00–1.03]; p = 0.03), altered mental status at admission (OR = 2.23; 95% CI [1.21–4.10]; p = 0.01) and seizures during the first 48 h of hospitalization (OR = 1.90; 95% CI [1.01–3.52]; p = 0.04) were independently associated with CVC (Table 3). In the sensitivity analysis restricted to CVC occurring after 48 h from admission, advanced age and altered mental status at admission remained independently associated with the occurrence of CVC (Table S1) whereas dexamethasone use was still not.

## Discussion

Our study shows that cerebrovascular complications were common, occurring in one quarter of adult patients with community-acquired bacterial meningitis. This high rate of CVC was in agreement with results reported by others [12, 14, 22]. In multivariate analysis, we identified advanced age, altered mental status and seizures during the first 48 h of hospitalization associated with occurrence of CVC but not dexamethasone use.

The COMBAT study is to date one of the largest multicenter cohorts of adults with community acquired bacterial meningitis diagnosed according to rigorous criteria, and which analyzes relatively rare events such as meningitis complicated by CVC. In addition, the prospective inclusion of cases of meningitis in the 69 French centers allows the collection of a representative sample of community acquired bacterial meningitis cases in France [20]. COMBAT patient characteristics closely resemble to those reported in other industrialized countries as previously described [20]. Distribution of causative microorganisms was consistent with that described in the literature [2–5] with *S. pneumoniae* and *N. meningitidis* involved in about 3/4 (76.3%) of all cases. In bivariate analysis, *S. pneumoniae* was more frequently identified in patients who developed CVC (62.9% vs. 50.7%) whereas *N. meningitidis* was associated with a low risk of CVC despite its well-known association with disseminated intravascular coagulopathy.

In the 265 patients with *S. pneumoniae* meningitis, 78 (29.4%) developed CVC. This is consistent with that reported in the literature where arterial stroke occurred in up to 30% of patients with pneumococcal meningitis, cerebral venous thrombosis in up to 9%, and intracerebral hemorrhage in up to 9%[23]. Delayed cerebral thrombosis, either arterial or venous, is a rare but devastating complication, involving a hyperinflammatory syndrome and extensive cerebral infarction, days to weeks after initial good recovery [24]. However, the comparison between studies is made difficult by the heterogeneity of the included populations (adults and children), the small sample sizes and the lack of detailed information about definition of neurological complications.

The advanced age of the patient is a well-known risk factor for CVC, apart from the situation of infectious meningitis.

Alteration of consciousness, measured by the Glasgow score, has been previously described as a major marker of the severity of inflammation in bacterial meningitis [25]. We showed that a Glasgow score of less than 14 was associated with CVC in multivariate analysis. This result was in line with those of the Dutch Meningitis Cohort Study, a prospective nationwide observational cohort study in the Netherlands, who found that decreased level of consciousness was independently associated with cerebral infarction in adults with bacterial meningitis [12]. Seizures during hospitalization has also been identified as a factor independently associated with the onset of CVC.

The role of corticosteroids in the occurrence of CVC has been discussed in the literature. Their benefit is known to reduce mortality by 15 to 30% in patients with acute bacterial meningitis [7, 26]. However, a post hoc analysis of the randomized controlled trial [7] showed that the beneficial effect of dexamethasone was due to a decrease in systemic complications, rather than to a reduction in neurological complications [26, 27]. We found that administration of dexamethasone in patients with acute bacterial meningitis had no significant effect on the onset of CVC, whether it was started before, at the same time or just after the antibiotic therapy initiation. This result was surprising in view of the inflammatory context of CVC. It can therefore be hypothesized that the recommended corticosteroid therapy is not effective on these complications. However, we did not have sufficient data to address this point with a more complete analysis of treatments chronology and the adequacy to the therapeutic recommendations in bacterial meningitis.

This study suffers from limitations. First, as it was an observational study, patients did not have systematic brain imaging according to a predetermined protocol, which may have led to an underestimation of the frequency of asymptomatic CVC. Second, the radiological data did not make possible the distinction between venous and arterial infarctions because of the non-systematically contrast-injected nature of the images. Third, there was no independent reading of brain imaging by a neuro-radiologist. Fourth, the size of the different subgroups (definite symptomatic/definite asymptomatic/probable) was too small to allow stratified multivariate analyses. Finally, we did not have data on cardiovascular risk factors and the precise date of onset of cerebrovascular complications, which that were collected only by period (before 48 h, after 48 h).

In conclusion, we found that CVC in adult patients with community-acquired bacterial meningitis were frequent. Advanced age, alteration of consciousness on admission and seizures during hospitalization were independent factors associated with the occurrence of CVC whether dexamethasone use was not. Large-scale studies are needed to evaluate the effects of higher dose of dexamethasone on CVC during bacterial meningitis in adults.

## Electronic supplementary material

Below is the link to the electronic supplementary material.

Supplementary Material 1

Supplementary Material 2

## Data Availability

The data-sets used and/or analysed during the current study are available from the corresponding author on reasonable request.
