# Supplementary material for: Cerebrovascular complications in patients with community-acquired bacterial meningitis: occurrence and associated factors in the COMBAT multicenter prospective cohort
Source: BMC Infect Dis. 2023 Jun 5;23:376. doi: 10.1186/s12879-023-08320-x (PMC10243062; doi:10.1186/s12879-023-08320-x)
Supplement: Supplementary file 1 — Supplementary Material 1 [file 12879_2023_8320_MOESM1_ESM.docx]

**Cerebrovascular complications in patients with community-acquired bacterial meningitis: occurrence and associated factors in the COMBAT multicenter prospective cohort**

**Table S1**: Factors associated with cerebrovascular complications excluding patients with symptomatic CVC already present at hospital admission or occurring during the 48 first hours in the COMBAT study; N= 443.

|  | Patients with CVC (N=55) | Patients without CVC (N=378) | P-value of  bivariate analysis | Multivariable odds ratio [95% CI] | P-value of  multivariable analysis |
| --- | --- | --- | --- | --- | --- |
| **Demographic data** |  |  |  |  |  |
| Age, years (Median, IQR) | 62 (50-72) | 56 (38-67) | <0.001 | 1.01 [1.00 – 1.03] | 0.03 |
| Male | 29/55 (52.7%) | 205/378 (54.0%) | 0.88 |  |  |
| **Predisposing conditions, concomitant infections** |  |  |  |  |  |
| Immunocompromised state* | 13/55 (23.7%) | 90/372 (24.2%) | 0.90 |  |  |
| Otitis/sinusitis | 17/55 (30.9%) | 106/366 (28.9%) | 0.75 |  |  |
| Pneumoniae | 8/55 (14.5%) | 35/366 (9.6%) | 0.23 |  |  |
| **Symptoms on presentation** |  |  |  |  |  |
| Altered mental status | 44/54 (81.5%) | 245/369 (66.4%) | <0.002 | 2.53 [1.30-4.90] | 0.007 |
| **Blood chemistry tests** |  |  |  |  |  |
| Leukocyte count (10^3^ cells/mm^3^) | 15.0 (9.0 – 19.8) | 15.3 (9.1 – 21.5) | 0.89 |  |  |
| Indexes of inflammation in the CSF |  |  |  |  |  |
| Leukocyte count (cells/mm^3^) | 985 (210-4300) | 1760 (367-5700) | 0.14 |  |  |
| CSF Protein (g/l) | 5.7 (3.0 – 8.4) | 3.9 (2.0 – 6.3) | <0.001 |  |  |
| CSF: blood glucose ratio (mmol/l) | 0.2 (0.05 – 1.6) | 0.8 (0.1 – 2.55) | 0.03 |  |  |
| **Intra-hospital complication in the first 48 hours** |  |  |  |  |  |
| Seizure | 7/53 (13.2%) | 29/368 (7.9%) | 0.19 |  |  |
| Extensive necrotic purpura | 5/55 (9.0%) | 40/369 (10.8%) | 0.81 |  |  |
| Disseminated intravascular coagulation | 6/54 (11.1%) | 25/365 (6.8%) | 0.26 |  |  |
| **Adjunctive dexamethasone** |  |  |  |  |  |
| Administered before antibiotic initiation | 8/55 (14.5%) | 40/367 (10.9%) | 0.87 |  |  |
| Administered at the same time as antibiotics | 21/55 (38.2%) | 130/367 (35.4%) |  |  |  |
| Administered after antibiotic initiation | 12/55 (21.8%) | 96/367 (26.2%) |  |  |  |
| No dexamethasone administered during hospitalization | 14/55 (25.4%) | 101/367 (27.5%) |  |  |  |
| **Brain imaging performed** | 53/55 (96.4%) | 308/365 (84.4) | 0.01 |  |  |
| **Microorganisms** |  |  |  |  |  |
| *Streptococcus pneumoniae* | 35/53 (66.0%) | 187/369 (50.7%) | <0.001 |  |  |
| *Neisseria meningitidis* | 9/53 (17.0%) | 94/369 (25.5%) |  |  |  |
| Other microorganisms | 9/53 (17.0%) | 88/369 (23.8%) |  |  |  |

* An immunosuppressive state is defined by the use of immunosuppressive drugs, the presence of asplenia, congenital immunodepression, acquired hypogammaglobulinemia, hepatic cirrhosis, metastatic cancer, malignant hemopathy, chronic or HIV infection.
