# Supplementary material for: Cerebrovascular complications in patients with community-acquired bacterial meningitis: occurrence and associated factors in the COMBAT multicenter prospective cohort
Source: BMC Infect Dis. 2023 Jun 5;23:376. doi: 10.1186/s12879-023-08320-x (PMC10243062; doi:10.1186/s12879-023-08320-x)
Supplement: Supplementary file 2 — Supplementary Material 2 [file 12879_2023_8320_MOESM2_ESM.docx]

**Combat study group**

Principal investigator: Xavier DUVAL

Steering Committee: Bruno HOEN, Bruno MOURVILLIER, Marie-Cécile PLOY, Sarah TUBIANA, Emmanuelle VARON.

Scientific committee: steering committee and the following members François CARON, Pierre-Edouard BOLLAERT, Olivier GAILLOT, Muhamed-Kheir TAHA, Claire POYART, Stephane BONACORSI, François VANDENESCH, Emmanuelle CAMBAU, Marc LECUIT, Alain GRAVET, Bruno FRACHET, Thomas De BROUCKER, Daniel LEVY BRUHL, François RAFFI, Marie PREAU.

COMBAT Clinical Centers: Nadia ANGUEL, Laurent ARGAUD, Sophie ARISTA, Laurence ARMAND-LEFEVRE, Stéphanie BALAVOINE, Régine BARADUC, Guilène BARNAUD, Guillaume BERAUD, Louis BERNARD, Georges BERNARS, Dominique BERTEI, Emilie BESSEDE, Typhaine BILLARD POMARES, Charlotte BIRON, Stéphane BLAND, Julien BOILEAU, Patrice BOUBEAU, Sandra BOURDON, Aurore BOUSQUET, Sophie BOYER, Alexis BOZORG-GRAYELI, Laurent BRET, Cédric BRETONNIERE, François BRICAIRE, Elsa BROCAS, Michel BRUN, Jennifer BURET, Christophe BURUCOA, Jean CABALION, Mathieu CABON, Emmanuelle CAMBAU, Guillaume CAMUSET, Christophe CANEVET, François CARON, Anne CARRICAJO, Bernard CASTAN, Eric CAUMES, Charles CAZANAVE, Amélie CHABROL, Thibaut CHALLAN-BELVAL, Vanessa CHANTEPERDRIX-MARILLIER, Chantal CHAPLAIN, Caroline CHARLIER-WOERTHER, Hélène CHAUSSADE Catherine CHIROUZE, Bernard CLAIR, Julien COLOT, Jean-Marie CONIL, Hugues CORDEL, Philippe CORMIER, Joël COUSSON, Pierrick CRONIER, Eric CUA, Anne DAO-DUBREMETZ, Sylvie DARGERE, Nicolas DEGAND, Sophie DEKEYSER, Deborah DELAUNE, Eric DENES, Pierre-Francois DEQUIN, Diane DESCAMPS, Elodie DESCLOUX, Jean-Luc DESMARETZ, Jean-Luc DIEHL, Jérôme DIMET, Aurélien DINH, Xavier DUVAL, Lelia ESCAUT, Claude FABE, Frédéric FAIBIS, Clara FLATEAU, Nathalie FONSALE, Emmanuel FORESTIER, Nicolas FORTINEAU, Amandine GAGNEUX-BRUNON, Caroline GARANDEAU, Magali GARCIA, Denis GAROT, Stéphane GAUDRY, François GOEHRINGER, Alain GRAVET, Valérie GREGOIRE-FAUCHER, Marine GROSSET, Camélia GUBAVU, Isabelle GUEIT, Dominique GUELON, Thomas GUIMARD, Jérôme GUINARD, Tahar HADOU, Jean-Pierre HELENE, Sandrine HENARD, Benoit HENRY, Anne-Cécile HOCHART, Bruno HOEN, Gabriela ILLES, Sylvain JAFFUEL, Irène JARRIN, Françoise JAUREGUY, Cédric JOSEPH, Marie-Emmanuelle JUVIN, Samer KAYAL, Solen KERNEIS, Flore LACASSIN, Isabelle LAMAURY, Philippe LANOTTE, Etienne LAURENS, Henri LAURICHESSE, Cécile LE BRUN, Vincent LE MOING, Paul LE TURNIER, Hervé LECUYER, Sylvie LEDRU, Céline LEGRIX, Adrien LEMAIGNEN, Chantal LEMBLE, Ludovic LEMEE, Olivier LESENS, Marion LEVAST, Claire LHOMMET, Silvija MALES, Edith MALPOTE, Guillaume MARTIN-BLONDEL, Matthieu MARX, Raphael MASSON, Olivier MATRAY, Aurore MBADI, Frédéric MECHAI, Guillaume MELLON, Audrey MERENS, Marie Caroline MEYOHAS, Adrien MICHON, Joy MOOTIEN YOGANADEN, David MORQUIN, Stéphane MOULY, Natacha MROZEK, Sophie NGUYEN, Yohan NGUYEN, Maja OGIELSKA, Eric OZIOL, Bernard PAGE, Solène PATRAT-DELON, Isabelle PATRY, André PECHINOT, Sandrine PICOT, Denys PIERREJEAN, Lionel PIROTH, Claire PLASSART, Patrice PLESSIS, Marie-Cécile PLOY, Laurent PORTEL, Patrice POUBEAU, Marie POUPARD, Claire POYART, Thierry PRAZUCK, Luc QUAESAET, François RAFFI, Adriatsiferana RAMANANTSOA, Christophe RAPP, Laurent RASKINE, Josette RAYMOND, Matthieu REVEST, Agnès RICHE, Stéphanie ROBADAY-VOISIN, Frédéric ROBIN, Jean-Pierre ROMASZKO, Florence ROUSSEAU, Anne-Laure ROUX, Cécile ROYER, Matthieu SAADA, Dominique SALMON, Carlo SAROUFIM, Jean Luc SCHMIT, Manuela SEBIRE, Christine SEGONDS, Valérie SIVADON-TARDY, Nathalie SOISMIER, Olivia SON, Simon SUNDER, Florence SUY, Didier TANDE, Jacques TANKOVIC, Nadia VALIN, Nicolas VAN GRUNDERBEECK, François VANDENESCH, Emmanuelle VARON, Renaud VERDON, Michel VERGNAUD, Véronique VERNET-GARNIER, Magali VIDAL, Virginie VITRAT, Daniel VITTECOQ, Fanny VUOTTO.

Coordination and statistical analyses (Clinical trial unit, Hôpitaux Universitaires Paris Nord Val de Seine, AP-HP, Paris) : Isabelle GORENNE, Cédric LAOUENAN, Estelle MARCAULT, France MENTRE, Blandine PASQUET, Carine ROY, Sarah TUBIANA.

Partners: ORP (Marie-Cécile PLOY), GPIP/ACTIV (Corinne Levy)
